# A rare malignant thyroid carcinosarcoma with aggressive behavior and DICER1 gene mutation: a case report with literature review

**DOI:** 10.1186/s13044-018-0055-8

**Published:** 2018-07-31

**Authors:** Jing Yang, Carmen Sarita-Reyes, David Kindelberger, Qing Zhao

**Affiliations:** Department of Pathology and Laboratory Medicine, Boston University Medical Center, 670 Albany St. Biosquare III, Boston, MA 02118 USA

**Keywords:** Thyroid, Carcinosarcoma, Biphasic tumor, Lung metastasis, DICER1 mutation

## Abstract

**Background:**

Malignant biphasic tumor also known as carcinosarcoma is an uncommon neoplasm that is composed of both malignant epithelial and mesenchymal components. Most reported cases of carcinosarcoma affect the female genital tract; however, other sites including head and neck, lung, and breast have been described. Carcinosarcoma of the thyroid is an extremely rare and aggressive malignancy with an ominous clinical course similar to anaplastic carcinoma.

**Case presentation:**

We report a case of a 45-year-old female who was found to have a biphasic thyroid carcinosarcoma. Her clinical course declined significantly shortly after she underwent a total thyroidectomy and she developed distant metastases to the lungs. Histopathological features of the primary and metastatic tumor were identical. The tumor is composed of an intimately intermixed epithelial component of poorly differentiated follicular thyroid carcinoma and a spindle cell sarcoma with rhabdomyosarcoma differentiation. Molecular analysis using a next-generation sequencing based assay revealed a *DICER1* (E1705K) point mutation in neoplastic cells.

**Conclusion:**

To our knowledge, the E1705K point mutation within the *DICER1* gene is the first reported mutation in carcinosarcoma of the thyroid. A comprehensive review of the relevant literature is also included for discussion.

## Background

Thyroid carcinosarcoma is a rare and aggressive malignant thyroid tumor [1]. These tumors are usually found to infiltrate the surrounding soft tissue at the time of diagnosis. The overall survival rate for these patients is only a few months and most of the cases occur in women who are older than 50 years of age. To date, less than 30 cases of thyroid carcinosarcoma have been reported in the literature (Table 1) [1–8]. According to the latest World Health Organization (WHO 2017) classification for thyroid tumors, carcinosarcoma is considered to be a variant of anaplastic carcinoma [9]. Recently, Agrawal et al. proposed that, with the presence of both malignant epithelial and mesenchymal cells, ‘thyroid carcinosarcoma’ should be considered a distinct entity from anaplastic carcinoma [6].

**Table 1:** Summary of all reported cases of thyroid carcinosarcoma in English literature

Author; year; Ref [#]	Morphology	Metastasis	Treatment	Survival (months)
Rasheed; 2017; [8]	Follicular carcinoma with sarcoma	none	total thyroidectomy, chemotherapy	3
Ekici; 2015; [7]	Papillary carcinoma with sarcoma	none	total thyroidectomy	2
Agrawal; 2013; [6]	Papillary carcinoma with sarcoma	lymph nodes	total thyroidectomy, right neck dissection	12
Naqiyah; 2010; [5]	Follicular carcinoma with sarcoma	none	total thyroidectomy, radiation	8
Guiffrida; 2000; [1]	Follicular carcinoma with sarcoma	bilateral lung, lymph nodes	total thyroidectomy lymph node dissection and chemoradiation therapy	6
Al-Sobhi; 1997; [4]	Follicular carcinoma with sarcoma	lung	total thyroidectomy, radiation	8
Cooper; 1989; [3]	Follicular carcinoma with chondrosarcoma	N/A	subtotal thyroidectomy	5
Donnell; 1987; [2]	Follicular carcinoma with osteosarcoma and chondrosarcoma	lung	subtotal thyroidectomy	26

The pathogenesis of thyroid carcinosarcoma is not fully understood. Carcinosarcoma of the thyroid has been suggested to originate from both malignant epithelial (carcinoma) and mesenchymal (sarcoma) elements of the thyroid [1, 6]. Positive immunohistochemical (IHC) staining for thyroglobulin in carcinomatous cells, and positive staining for vimentin in mesenchymal cells supports a diagnosis of carcinosarcoma [1]. In contrast, studies from anaplastic thyroid carcinoma suggested a monoclonal origin of the tumor [2]. The sarcoma-like morphology of the tumor is thought to be de-differentiated from the epithelial component during the process of carcinogenesis.

In 2013, Agrawal et al. reviewed 25 cases reported in the literature as carcinosarcoma of the thyroid. Since then, there have been two additional cases reported. Among the reported cases, most were described as fibrosarcoma or osteosarcoma with co-existing differentiated thyroid carcinoma, such as follicular or papillary carcinoma [1–8]. In most of the cases, the tumors behaved aggressively, with death from disease occurring within 2 months to 26 months following diagnosis [1–8]. No specific molecular signatures or genetic alteration have been reported. The current report presents a case of a young female patient with a biphasic thyroid carcinosarcoma. Next generation sequencing (NGS) analysis revealed a novel point mutation in the *DICER1* gene (E1705K) in neoplastic cells.

## Case presentation

A 45-year-old woman presented to our hospital with multiple lung nodules. She had a history of poorly differentiated thyroid carcinoma, diagnosed 7 months prior to admission, at an outside hospital. The patient was healthy otherwise and reported no radiation exposure or any family history of thyroid cancer. The initial work-up at the time of discovery of the right thyroid nodule included fine needle aspiration and core biopsy, with findings consistent with poorly differentiated thyroid carcinoma. The patient then underwent a total thyroidectomy and central neck lymph node dissection. The pathologic diagnosis from the outside hospital reported a 2.8 × 2.4 × 1.1 cm tumor in the right thyroid without extrathyroidal extension or lymph node metastasis. However, both capsular invasion and extensive vascular space invasion were noted. Based on the tumor size, tumor extension and lymph node status, the tumor was designated as Stage II (pT2 pN0 pMx). IHC staining showed that the tumor cells were positive for thyroglobulin and thyroid transcription factor 1 (TTF1). An immunostain for p53 was also performed at the outside hospital and showed a small focus (< 1 cm) with p53 positivity, suggesting a diagnosis of anaplastic thyroid carcinoma.

**Fig. 1:**
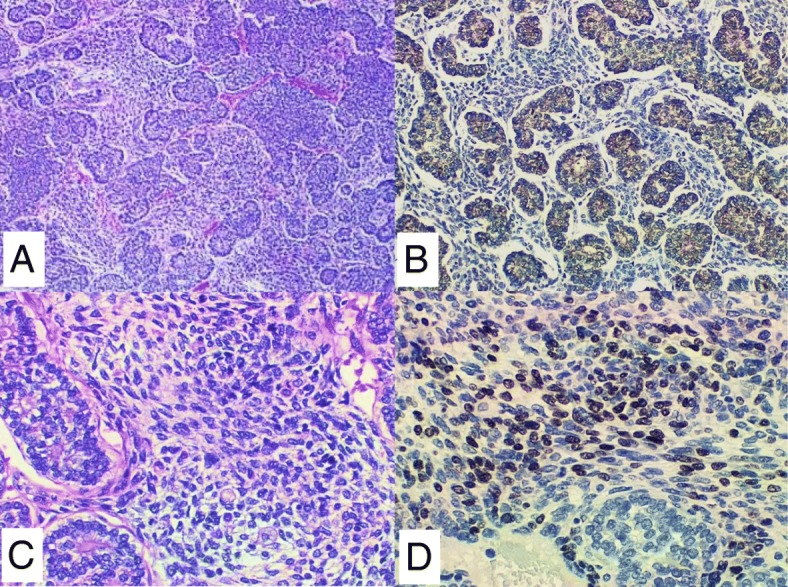
Immunohistochemical stains demonstrated biphasic components. The carcinoma component (**a**) showed positivity for thyroglobulin (**b**). The sarcoma component (**c**) showed positivity for myogenin (**d**). All pictures are at 200×

At our institution, the diagnosis was revised, based on review of both the primary thyroid tumor and the current lung metastases. Both tumors were remarkable for biphasic malignant components: the carcinoma and the sarcoma. The carcinoma component showed a poorly differentiated microfollicular type thyroid carcinoma, composed of sheets and islands of tightly packed thyroid follicles with dense colloid. The tumor nuclei were small and round with vesicular chromatin, resembling those of typical poorly differentiated follicular thyroid carcinoma. Admixed with the epithelial component were malignant spindle cells with small round blue cell type morphology. Focally, rhabdomyosarcoma-like cells with eosinophilic cytoplasm were appreciated. No heterologous cartilage or bone components were identified. The IHC staining performed at the outside hospital showed that the thyroid carcinoma (epithelial) component was positive for thyroglobulin, PAX8 and TTF1 (Fig. 1). The sarcoma (spindled) component was negative for all thyroid carcinoma markers (TTF-1, thyroglobulin and PAX8), but was positive for vimentin and focally positive for myogenin (supporting skeletal muscle differentiation) consistent with mesenchymal differentiation. Interestingly, the foci of vascular space invasion contained both epithelial and mesenchymal components as well.

The patient received Taxol with Carboplatin for 7 weeks followed by radiation therapy. Her thyroglobulin level rose from 1.2 ng/mL to 25.40 ng/mL 5 months after completion of the chemo-radiation therapy, suggesting progression of the disease. A follow-up CT scan of the chest showed multiple newly developed nodules (ranging from 1 to 2 cm) in the right lung, highly suspicious for metastases. The patient underwent a right thoracotomy, right lung resection/metastasectomy. The surgery was uneventful with negative resection margins. However, the patient’s general condition deteriorated and she succumbed to the disease 4 months later.

**Fig. 2:**
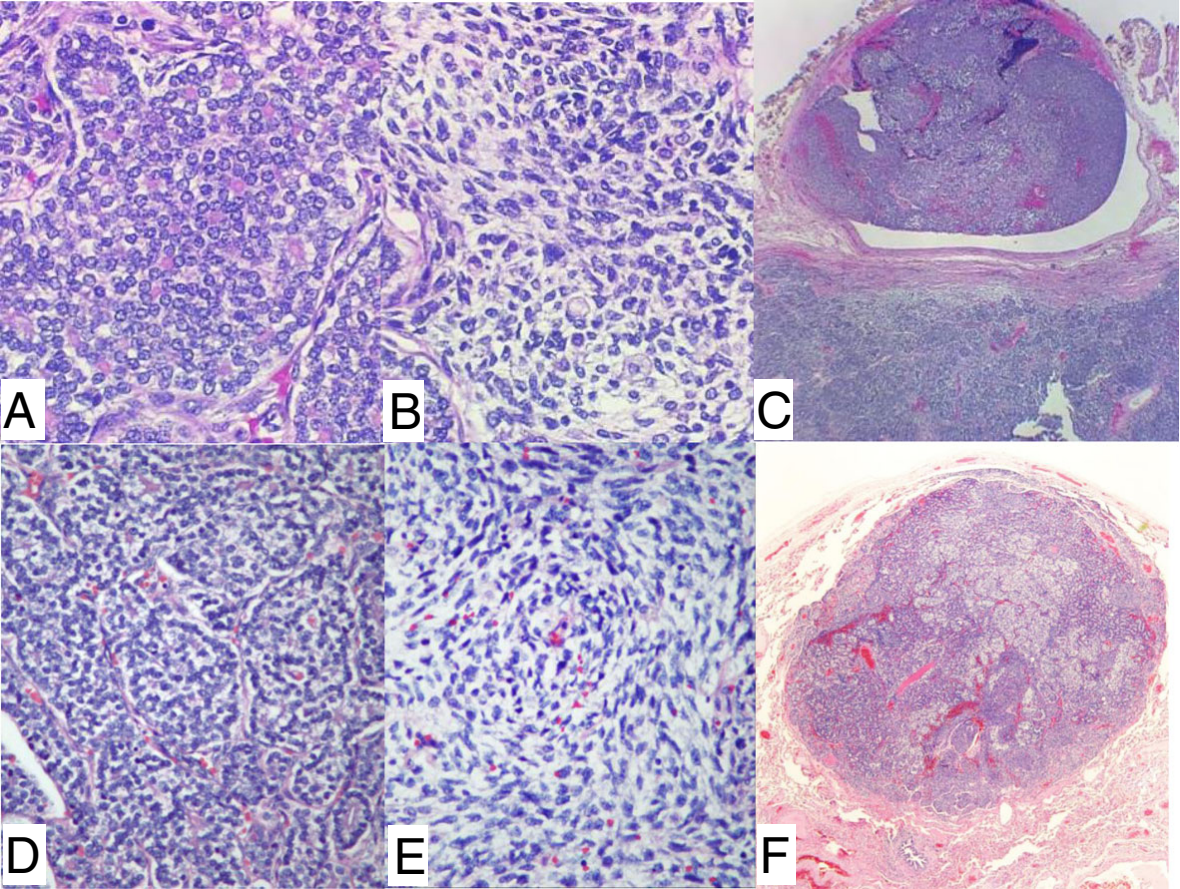
Histological features of primary thyroid cancer and metastatic lung nodules. The epithelial component in the thyroid cancer (**a**) is morphologically similar to the epithelial component in the lung nodule (**d**). The sarcomatous component is composed of spindle cells both in the thyroid cancer (**b**) and in the lung nodule (**e**). There is vascular invasion in the thyroid cancer (**c**). Low power review of a small lung nodule (40×) in (**f**)

Histological examination of the lung nodules revealed similar tumor morphology and tumor differentiation when compared to the original thyroid tumor, which is somewhat unusual for a biphasic carcinosarcoma (Fig. 2). Tumor necrosis was also present. Mutational analysis using a next-generation sequencing based assay showed that the neoplastic cells from the lung metastasis were devoid of genomic alterations for known thyroid cancers, including *BRAF*, *RAS* family (*KRAS, NRAS* and *HRAS*), *EGFR*, *PTEN*, *TERT*, *PI3Kinase* or *RET*. *BRAF* or *RAS* family are known as the most commonly altered genes in papillary thyroid cancers. Other molecular mutations reported in the development of anaplastic thyroid carcinoma include p53, *PAX8/PPAR* gamma rearrangement [10]. None of the mentioned gene mutations were identified in our patient.

However, an interesting finding in this case is the presence of a point mutation in *DICER1* (E1705K*)* that has previously been associated with differentiated thyroid carcinoma [11, 12]. Whether the *DICER1* (E1705K) mutation is the underlying genetic event leading to the initiation of tumorigenesis or is downstream to other gene alterations in tumor development is largely unknown. Additional mutations of unknown significance were also detected in this tumor including *FLCN* (R239H), *POLD1* (Q684H) and *SYK* (R217L). These variants have not been adequately characterized in the scientific literature and their prognostic and therapeutic significance is unclear.

## Discussion and conclusions

Thyroid carcinosarcoma is a very aggressive malignant tumor with a clinical course similar to that of anaplastic thyroid carcinoma [4]. All reported thyroid carcinosarcoma cases have resulted in patients only surviving a few months after initial diagnosis [1–8]. To our knowledge, there is no previously published molecular analysis of thyroid carcinosarcoma. This is the first report to describe potential gene alterations identified using next generation sequencing. The DICER1 protein is a member of the ribonuclease III (RNase III) family that plays an important role in the post-transcriptional regulation of gene expression. Heterozygous germline *DICER* mutations are described in the so-called DICER1 syndrome, which leads to a predisposition to develop a variety of tumors in children, including pleuropulmonary blastoma, cystic nephroma, rhabdomyosarcoma, ovarian Sertoli-Leydig tumors and multinodular goiter. A recent case study reported that a germline *DICER1* mutation (S1814 L) is associated with an increased risk of thyroid follicular carcinoma [11]. A somatic *DICER1* hotspot mutation at E1705K was reported in a case of anaplastic sarcoma of the kidney and in approximately 60% of Sertoli-Leydig cell tumors [12, 13]. The presence of this point mutation of *DICER1* at E1705K in thyroid carcinosarcoma has never been reported. The presence of a *DICER1* mutation in this case provides further evidence that development of the carcinosarcoma may not be a random occurrence, but is a likely due to a specific genetic alteration. Due to the highly aggressive nature of thyroid carcinosarcomas, the identification of specific genetic signatures for these tumors becomes critical in identifying effective treatments. However, there are still many questions regarding the pathogenesis and tumorigenesis of thyroid carcinosarcoma. Further studies will be of great benefit for the diagnosis and treatment.

Other mutations that were identified in this tumor include *POLD1* (Q684H), *FLCN* (R239H) and *SYK* (R217L). *POLD1* (Q684H) is a recently described mutation that had been reported in a patient with colorectal cancer [14]. Germline mutations in the folliculin (FLCN*)* gene, encoding the folliculin tumor-suppressor protein, are reported to be associated with Birt Hogg Dubé syndrome [15]. Spleen tyrosine kinase (SYK) is an essential enzyme required for signaling involving multiple classes of immune receptors [16]. SYK functions as a modulator of tumorigenesis and has been reported in association with leukemia and breast cancers [16]. The clinical significance of *FLCN* (R239H) and *SYK* (R217L) mutations in thyroid carcinosarcoma is unknown.

The differential diagnosis of thyroid carcinosarcoma includes anaplastic carcinoma. P53 point mutations are present in 60–80% of anaplastic thyroid carcinomas. Patients develop anaplastic carcinoma from a pre or coexistent differentiated carcinoma after a multistep process of dedifferentiation associated with loss of the p53 oncogene suppressor. Due to the similarities between anaplastic carcinoma and carcinosarcoma of the thyroid gland, it has been proposed that p53 mutation may contribute to the pathogenesis of carcinosarcoma. In our patient, a less than 1 cm focus within the anaplastic area showed p53 IHC positivity. No P53 mutation was detected by next generation sequencing. Molecular pathogenetic mechanisms of p53 involvement in the transformation of carcinosarcoma are not well understood. Other mutations reported in anaplastic thyroid carcinoma include *RAS, BRAF, PTEN, TERT,* and PIK3kinase which were not identified in this patient. Thus, the diagnosis of anaplastic carcinoma is not supported by our studies.

The clinical course of thyroid carcinosarcoma is similar to anaplastic carcinoma. Some case reports have recommended following the standard treatment approach for anaplastic carcinoma. Multimodality treatment for anaplastic carcinoma, consisting of radical surgery followed by radiotherapy and chemotherapy is reported to be associated with better clinical outcomes. However, there is no uniform consensus about the treatment approach for thyroid carcinosarcoma due to its very low incidence, its aggressive nature with poor prognosis, and the consequent lack of large clinical series. Most reported cases were treated with total or subtotal thyroidectomy. Adjuvant chemotherapy, radiation therapy and immunotherapy have not proven to be beneficial. However, NGS performed on collected thyroid carcinosarcoma cases could be of great benefit for the identification of targeted treatments.

In conclusion, the present study reports a rare case of primary thyroid carcinosarcoma with metastasis to the lung in a 45-year-old female patient who ultimately succumbed to the disease after receiving surgeries for primary and metastatic tumors and adjuvant chemoradiation therapy. Her total survival time was 11 months from the time of diagnosis and her total disease free survival was 7 months. A *DICER1* (E1705K) gene mutation was identified in this patient. Primary thyroid carcinosarcoma is extremely rare and can be diagnostically challenging. Immunohistochemical staining may be useful for establishing a diagnosis and for distinguishing the disease from anaplastic carcinoma. Although the overall survival is dismal despite aggressive treatment, careful evaluation and the use of NGS to detect specific gene alterations may lead to the development of effective targeted therapies.

## Abbreviations

BRAF: B-Raf Proto-Oncogene
DICER1: Ribonuclease III
EGFR: Epidermal growth factor receptor
IHC: Immunohistochemical
NGS: Next generation sequencing
PAX8: Paired box gene 8
PIK3kinase: Phosphatidylinositol-4, 5-bisphosphate 3-kinase
RET: Proto-oncogene encodes a receptor tyrosine kinase
TTF1: Thyroid transcription factor 1
WHO: World health organization
